# Effects of salpingectomy during abdominal hysterectomy on ovarian reserve: a randomized controlled trial

**DOI:** 10.1186/s10397-017-1019-z

**Published:** 2017-08-17

**Authors:** Afsaneh Tehranian, Roghayeh Hassani Zangbar, Faezeh Aghajani, Mahdi Sepidarkish, Saeedeh Rafiei, Tayebe Esfidani

**Affiliations:** 1 Department of Obstetrics and Gynecology, Roointan-Arash Women’s Hospital, Tehran University of Medical Sciences, Tehran, Iran; 2 Department of Epidemiology and Reproductive Health, Reproductive Epidemiology Research Center, Royan Institute for Reproductive Biomedicine, ACECR, Tehran, Iran

**Keywords:** Salpingectomy, Hysterectomy, Ovarian reserve, Anti-Mullerian hormone

## Abstract

**Background:**

The aim of this study was to investigate the effect of salpingectomy on ovarian function by measuring AMH.

**Methods:**

This study was a balanced, single-center, double-blind, randomized, controlled trial in Ruin Tan Arash Hospital, Tehran, between May 2013 and November 2014. A total of 30 patients undergoing elective abdominal hysterectomy were randomized into two groups, 15 with salpingectomy and 15 without salpingectomy. The primary objective of this study was to compare mean difference of anti-Mullerian hormone (AMH) between two groups. The secondary outcomes measured were follicle-stimulating hormone (FSH), operative time, and blood loss.

**Results:**

Serum AMH levels decreased at 3 months after hysterectomy in all patients (pre AMH 1.32 ± (0.91); post AMH 1.05 ± (0.88), *P* < 0.001), the salpingectomy group (pre AMH 1.44 ± (0.94); post AMH 1.13 ± (0.86), *P* < 0.001), and no salpingectomy group (pre AMH 1.2 ± (0.9); post AMH 0.97 ± (0.92), *P* < 0.001). The rate of decline of AMH levels after surgery did not differ between the two groups (25% (17–33%) vs. 26% (15–36%), *P* = 0.23) among the women with salpingectomy versus without salpingectomy, respectively. There was no difference in the mean operative time (mean difference 0.33, 95% CI − 22.21 to 22.86, *P* < 0.92), mean blood loss (mean difference − 0.66, 95% CI − 15.8 to 14.46, *P* < 0.97), and post FSH (mean difference 0.34, 95% CI − 1.2 to 1.88, *P* < 0.65) between both groups.

**Conclusions:**

Salpingectomy with abdominal hysterectomy is a safe treatment that does not have a deleterious effect on ovarian reserve.

**Trial registration:**

Iranian Registry of Clinical Trials, IRCT2014123118866N4 (www.IRCT.ir)

## Background

Hysterectomy is one of the most common surgeries in women worldwide [1]. It is applied for the treatment of various problems, such as pelvic pain, menstrual problems, tumors, and other related diseases. However, based on the patient’s problem, in addition to the uterus, removal of the fallopian tubes, ovaries, or cervix may be necessary [2]. Every year, 600,000 women are undergoing hysterectomy surgery in the USA [3]. The surgery is done as abdominally and vaginally, but from 1980 onwards, it is also done by laparoscopy, which plays a major role in the treatment of gynecologic malignancies, uterine leiomyoma, endometrial hyperplasia, and uterine prolapse [3, 4].

Hysterectomy may have complications. One of the most important complications is reduced ovarian function, which is not dependent on the type of surgery and is very important for women of reproductive age [1, 5]. Previous studies have shown that women undergoing hysterectomy experience menopausal symptoms faster and compared with other women have lower number of follicles, lower serum progesterone levels, and higher levels of follicle-stimulating hormone (FSH) [6]. The measures for preserving ovarian function after hysterectomy are always important.

Salpingectomy is a procedure for sterilization and with hysterectomy leads to good results, especially in recent decades [7, 8]. Salpingectomy can be due to different reasons, including treatment of ectopic pregnancy, infections in the fallopian tubes, and fallopian tube prolapse treatment after hysterectomy [9]. It seems that preserving ovarian function after hysterectomy is very important. Hysterectomy preserves the both ovaries and tubes through salpingectomy close to the uterus to preserve blood supply to the mesosalpinx of ovaries [10]. Many gynecologists refuse to perform salpingectomy at the time of hysterectomy due to blocking uterine blood flow to the ovaries and disrupting its function [11]. There is no agreement on the effect of salpingectomy, and some studies revealed the devastating impact of salpingectomy [12]. Interestingly, findings of studies have shown that the primary source of ovarian cancer is fallopian tubes and if hysterectomy is along with salpingectomy, cancer progression may be prevented. The preferred surgery is removing tubes associated with hysterectomy in women who have high levels of uterine cancer [13]. It can be proved in practice that hysterectomy with salpingectomy has no harmful effects on ovarian function and is used as a suitable technique for hysterectomy. The aim of this study was to investigate the effect of salpingectomy on ovarian function by measuring anti-Mullerian hormone (AMH) (as a substitute for ovarian reserve). Therefore, the mean AMH was compared between the two groups of hysterectomy and hysterectomy with salpingectomy within 3 months after surgery.

## Methods

This study was a balanced, single-center, double-blind, randomized, controlled trial in Ruin Tan Arash Hospital, Tehran, between May 2013 and November 2014.

The participants were premenopausal women aged 18 to 45 years who were undergoing abdominal hysterectomy for non-malignant gynecologic disease with preservation of the ovaries. All patients gave written informed consent before any study-related tests were done. Ethics approval was obtained from the Tehran University of Medical Sciences Clinical Research Ethics Board. Date of approval was January 31, 2015. The study was registered in Iranian Registry of Clinical Trials (www.IRCT.ir) by the number of IRCT2014123118866N4.

The inclusion criteria were age < 45 years, elective hysterectomy (without oophorectomy), absence of menopausal symptoms, and baseline FSH value of <10 IU/mL. Women with the following characteristics were excluded prior to enrollment: a history of pelvic surgery, cystic (> 10 mm) or any solid ovarian mass in transvaginal ultrasound, hormone replacement treatment and/or hormonal contraception for the last 6 months, a history of pelvic surgery, and a present or past smoking history.

After their consent was obtained, patients were randomly assigned in two groups, using a random number sequence, generated with a proprietary computer application, according to a randomized block design. The randomization list was created by the clinical trial’s epidemiologist, who kept the codes until completion of the study. Allocation concealment was maintained by having procedure indicator cards inside a set of numbered opaque sealed envelopes. Patients were allocated to treatment by the author opening the next numbered envelope, after screening, in the presence of the patient. None of the staff or patients had access to the randomization codes during the study. A patient’s treatment assignment would only be unblended when knowledge of the treatment was essential for the further management of the patient. The surgeon performing the procedures was blinded to the treatment allocation until the time of surgery.

Group 1 patients (salpingectomy) underwent total hysterectomy with removal of the fallopian tubes bilaterally. Caution was given to avoid injury to the ovarian vessels and to divide the mesosalpinx as close to the fallopian tube as possible. In group 2 (without salpingectomy), the fallopian tubes were divided in the proximal tubal isthmus.

The primary objective of this study was to compare mean difference of anti-Mullerian hormone (AMH) between two groups. The secondary outcomes measured were follicle-stimulating hormone (FSH), operative time, and blood loss.

The serum samples were collected preoperatively and at 3 months after the surgery from each patient. All hormonal measurements were performed in the same reference laboratory. Blood samples were obtained by venipuncture, and the sera extracted by centrifuge. Serum FSH and LH levels were measured by enzyme-linked fluorescent assay (VIDAS, BioMerieux SA) according to the manufacturer’s instructions. Serum AMH level was measured by enzyme-linked immunosorbent assay (ELISA) kit according to the manufacturer’s instructions (AMH Gen II ELISA; Immunotech) and reported as nanograms per milliliter with the detection limit of 0.006 ng/mL. The intraassay and interassay coefficients of variation (CV) were 4.38 and 5.64% for FSH, 4.14 and 4.86% for AMH, and 4.23 and 5.48% for LH, respectively.

The study required the enrollment of 30 patients in each group to have at least 80% power to detect mean difference of 0.5 between two groups with regard to main outcome (with two-sided test and type 1 error of 5%).

All analyses were performed on an intent-to-treat basis. Summaries of continuous and categorical measures were presented as the mean± (SD) and *N* (%) respectively. We compared a difference between baseline characteristics of patients and after randomization into the two groups with a chi-square test for categorized data and with Student’s *t* test for continuous variables. GLM (general linear model) (family = Gaussian, link = identity) was used to compare the two study arms for the primary and secondary end point at 3 months after the surgery. The model included treatment as main effects and age, body mass index (BMI), pre AMH, parity, pre FSH, and pre LH as covariates. Testing was performed at a 95% significance level. Results were presented as the mean difference with 95% confidence intervals. Statistical tests were two tailed. Data were analyzed using Stata software version 13 (Stata Corp, College Station, TX, USA). The conduct and analysis of the trial adhered to the 2010 CONSORT guidelines.

## Results

**Fig. 1:**
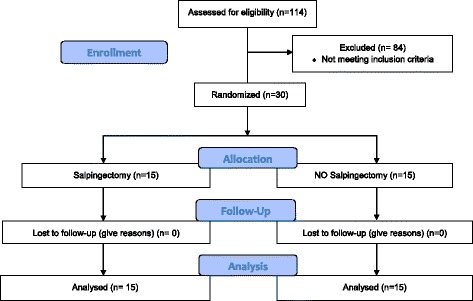
Flow diagram of the study selection process

In this study, 114 patients were recruited between January 2, 2014 and November 3, 2014. Eighty-four patients did not meet the criteria for participation. Ultimately, 30 patients were randomized into two groups: 15 women with salpingectomy and 15 women without salpingectomy. There were no complications directly attributable to performing salpingectomy. The study profile is shown in Fig. 1.

The mean age of patients was 40.13 (95% CI 38.88–41.38). The mean BMI of participants was 28.22 (95% CI 26.86–29.58) kg/m2 and mean uterine weight of 289.66 (95% CI 233.71–345.61) grams. Mean age in the salpingectomy group was 39.08 years (SD 3.72) and 40.46 years (SD 3.02) in the without salpingectomy group. Baseline and surgical characteristics in two groups are described in Table 1. The study groups were well matched with respect to demographics and disease characteristics.

**Table 1:** Baseline demographics and clinical characteristics

	Salpingectomy (*n* = 15)	No salpingectomy (*n* = 15)	*P* value
Age (years)	39.8 ± (3.72)	40.46 ± (3.02)	0.59
BMI	27.51 ± (4.14)	28.94 ± (3.02)	0.29
Parity	3.13 ± (0.51)	2.86 ± (0.99)	0.36
Pre AMH	1.2 ± (0.9)	1.44 ± (0.94)	0.47
Pre FSH	7.48 ± (1.84)	7.21 ± (2.48)	0.74
Pre Hb	12.26 ± (0.91)	11.82 ± (1.06)	0.24
Diameter ovarian	12.26 ± (1.09)	12.06 ± (1.16)	0.63
Uterine weight (g)	263.33 ± (118.12)	316 ± (176.26)	0.34

**Fig. 2:**
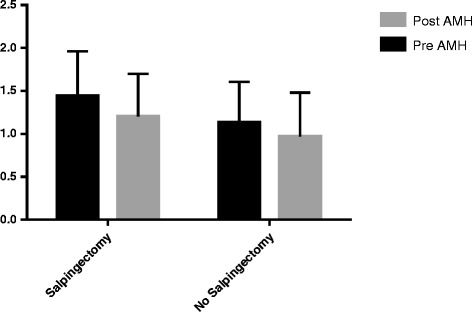
The rate of decline of AMH levels after surgery among the women with salpingectomy vs no salpingectomy

Serum AMH levels were decreased at 3 months after hysterectomy in all patients (pre AMH 1.32 ± (0.91); post AMH 1.05 ± (0.88), *P* < 0.001), the salpingectomy group (pre AMH 1.44 ± (0.94); post AMH 1.13 ± (0.86), *P* < 0.001), and without salpingectomy group (pre AMH 1.2 ± (0.9); post AMH 0.97 ± (0.92), *P* < 0.001). The rate of decline of AMH levels after surgery did not differ between the two groups (25%(17–33%) vs. 26%(15–36%), *P* = 0.23) among the women with salpingectomy versus without salpingectomy respectively (Fig. 2). Also in multivariate analysis, there was no significant difference between two groups at 3 months after operation (mean difference 4.46, 95% CI − 0.19 to 0.04, *P* < 0.21).

There was no difference in the mean operative time (mean difference 0.33, 95% CI -22.21 to 22.86, *P* < 0.92), mean blood loss (mean difference − 0.66, 95% CI − 15.8 to 14.46, *P* < 0.97), and post FSH (mean difference 0.34, 95% CI − 1.2 to 1.88, *P* < 0.65) between both groups.

## Discussion

The findings of this study confirmed the results of previous studies that showed that salpingectomy with hysterectomy cannot cause to a devastating effect on the ovarian reserve and 3 months after treatment, no significant difference was seen in the levels of AMH between the two treatments. Also, no significant differences were observed between the two groups in terms of surgical time and blood loss, and compared with hysterectomy, salpingectomy was not associated with any complications [14–16].

In a study by Sezik et al. in 2007, 24 patients were randomly divided into two groups (12 subjects in each group). In one of the groups, hysterectomy was performed with removal of both tubes, but in another group, the tubes were partially removed. One and 6 months after the intervention, patients were compared according to hormonal profile. The mean levels of hormones FSH, LH, estradiol, and ovarian volume were similar in both groups [14]. Morelli et al. in 2013 compared 158 patients retrospectively. A group of patients underwent hysterectomy without salpingectomy, and another group was women who had hysterectomy with salpingectomy**.** In the present study, no significant difference was observed between the two groups based on the levels of hormones AMH, FSH, antral follicle count, and the mean ovarian volume and peak systolic velocity was same in both groups [15]. A pilot study was conducted in North Carolina on 30 women aged 18 to 45 years were under laparoscopic hysterectomy along with bilateral salpingectomy or without salpingectomy. The AMH level was measured 3, 4 and 6 months after the intervention and then compared between the two groups. A significant decrease was not seen within groups at the baseline in AMH levels compared with after the intervention. Also, there was no significant difference between the two groups after intervention in both measurement times [17].

In keeping with the findings of the study, mean operative time and blood loss between the two groups were not significantly different. Although consistent results were obtained in line with other studies, contrary to the findings several studies reported an adverse effect of salpingectomy on the ovarian reserve [18–20]. In a retrospective study by Xu-ping Ye et al. on 198 women who were eligible for IVF-ET (in vitro fertilization—embryo transfer), AMH levels were compared among three groups of unilateral salpingectomy (83 subjects), bilateral salpingectomy (41 subjects), and no tubal surgery (54 subjects). The mean AMH in the group without tubal surgery was higher than the bilateral salpingectomy group. (183.48 vs. 127.11 fmol/ml, *P* = 0.037). The mean FSH was significantly higher in the bilateral salpingectomy group than the group without tubal surgery (7.85 vs 9.13 mLU/ml, *P* = 0.048) [21]. Findings from consequences of 288 IVF-ET cycles in 251 women with tubal factor infertility from January 2001 to December 2011 revealed that there was no significant difference between none ovarian response parameters in the two groups with and without salpingectomy [10].

The disagreement among studies can be attributed to the following reasons. One of the reasons may be due to different designs in these studies. Given that the best evidence comes from randomized clinical trial (RCT), more information can be cited from this type of studies [22]. Of abovementioned studies, one of them was RCT and others were retrospective cohort and case-control. The present study was conducted as RCT with relatively satisfactory sample size that confirmed the results of others studies and did not report a harmful effect of salpingectomy. Another reason was the use of different markers of ovarian reserve in the studies [22]. In the retrieved studies, reported measures included duration of gonadotropin stimulation, the amount of used gonadotropin, follicle count, the number of retrieved oocytes, fertility rate, and hormone levels (FSH, LH, estradiol, and AMH).

AMH is a glycoprotein dimer mainly secretes from granulocytes of preantral and small antral follicles. AMH levels are relatively constant throughout the menstrual cycle and have a very strong correlation with the number of follicles and ovarian reserve and are an important indicator of fertility [23, 24]. Previous studies have shown that AMH compared with other hormone markers is better predictive factor and is less affected by manipulating the endogenous gonadotropin. In the present study, AMH measured best predict ovarian reserve [25].

Some studies have shown that an effect of salpingectomy is harmful in infertile patients undergoing IVF-ET. In these studies, confounding factors affecting ovarian reserve and the process of infertility treatment (eligibility for IVF) were not adjusted. Infertile individuals undergoing ART is specific and cannot be a representative of all women of reproductive age [10]. Therefore, the results of these studies can be influenced by selection bias. Another factor that could contradict the results of these studies is due to measurement bias the skill of the surgeon.

It seems that if salpingectomy is done with minimum damage to the ovarian microvascularization, blood flow to the ovaries will be complete and the ovary function remains undisturbed. Adequate amounts of blood flow have a vital role in the follicular maturation, either spontaneously or stimulated, by influencing the synthesis of steroid hormones [18]. Ovarian blood flow is provided from two basic sources: ovarian artery originated from the aorta and ramus ovaricus that comes from the uterine artery. The blood flow of the tubes arises from ovarian artery and ramus ovaricus. Studies that found harmful effect of salpingectomy concluded based on decreased blood flow to the ovaries and reduction in its efficiency [12]. One of the important reasons for the phenomenon is expertise and skill of the surgeons.

The strengths of this study included study design (RCT), measuring objective outcomes and high prediction, and relatively desirable sample size. The most important limitation of the study was lack of long-term follow-up that causes to no tracking medium-term and long-term effects of hysterectomy and salpingectomy in menopause patients and their ovarian function. The lack of uterine blood flow measurement is the limitations of this study.

## Conclusions

Salpingectomy with abdominal hysterectomy is a safe and convenient treatment that does not have a deleterious effect on ovarian reserve. It is suggested that multicenter RCT studies with higher sample size and longer duration of follow-up are done to extract more accurate and reliable results about the effects of salpingectomy on fertilization.

### Key message

The findings of this study confirmed the results of previous studies that showed that salpingectomy with hysterectomy cannot cause to a devastating effect on the ovarian reserve and 3 months after treatment, no significant difference was seen in the levels of AMH between the two treatments.
